# The Antiviral and Antimalarial Prodrug Artemisinin from the *Artemisia* Species: A Review

**DOI:** 10.3390/cimb46110718

**Published:** 2024-10-28

**Authors:** Gitishree Das, Han-Seung Shin, Jayanta Kumar Patra

**Affiliations:** 1 Research Institute of Integrative Life Sciences, Dongguk University-Seoul, Goyang-si 10326, Republic of Korea; gdas@dongguk.edu; 2 Department of Food Science and Biotechnology, Dongguk University-Seoul, Goyang-si 10326, Republic of Korea

**Keywords:** artemisinin, prodrug, *Artemisia annua*, antiviral, antimalarial, derivatives

## Abstract

Artemisinin is a truly fascinating drug in many ways. Since the unrestrained procedure of its detection, as an antimalarial drug, artemisinin has received a great deal of consideration. Recently, application of artemisinin-based combination therapy has been broadly applied for treating numerous ailments. Moreover, as an antimalarial compound, artemisinin and its associated compounds have abundant healing efficacy and can be repurposed for additional symptoms, like autoimmune infections, cancer, and viral contaminations. Recently a number of studies have highlighted the significance of the artemisinin-related compounds in SARS-CoV-2 treatment. The current review purposes to present a concise account of the history of the antiviral and antimalarial prodrugs—Artemisinin, from the Artemisia species. It is followed by its antiviral, antimalarial prospective, chemical nature and extraction procedure, photochemistry, mechanism of action, and its clinical trials and patents, and accentuates the significance of the mechanistic studies concerned for therapeutic results, both in viral and malarial circumstances.

## 1. Introduction

Plants have been the object of exploration for a long time, due to their exceptional properties. Recently, there has been an increasing demand in the search for natural drugs and medicines of plant origin, considering the side effects and toxicity aroused by the use of synthetic drugs and medicines [1]. There are plentiful plant secondary metabolites that have abundant, naturally active, valuable properties and deliver defense to the host plants from various predators including insects, bacteria, and virus [2]. The ancient medicinal practitioners value numerous medicinal herbs for their healing abilities, and about 40% of modern drugs are derived from plant origin [2,3,4].

All over the world, the genus Artemisia comprises around 500 species, distributed through numerous regions [5,6]. The artemisinin compound is an oxygenated sesquiterpene lactone typically made in the glandular trichomes of *Artemisia annua* L. plant (sweet wormwood) and considered to be exclusively accountable for the strong activity against the parasite’s blood stages [7,8,9,10]. Out of all the secondary metabolites present in plants, the lactone derivative artemisinin is considered to be very favorable, due to its various pharmacological potentials [2,11,12,13]. It is considered an interesting drug, on account of its discovery relating to the Chinese system of traditional medicine and its incredible effectiveness and influence as an antimalarial drug [2,14]. Currently, the production of artemisinin and its incorporation into the management of new infections has been broadly applied [15]. Recently, several studies have highlighted the significance of the artemisinin-related compounds in SARS-CoV-2 treatment [16]. It has been reported that the SARS-CoV-2 contagion starts as a slight respiratory tract infection followed by lung injury, oxidative stress, inflammatory conditions, multi-organ failure, and neurological issues [17,18,19]. The use of artemisinin-related drug candidates as an antioxidant and anti-inflammatory compound could be useful in blocking tissue fibrosis and, together with its safety and low-toxicity nature, makes it an admirable drug candidate against the SARS-CoV-2 infection [16,20]. The practice of artemisinin-based combination therapies (ABCTs) in the management of malaria has also been reported previously [7,9,21]. Moreover, as an antimalarial compound, artemisinin and its associated compounds have abundant healing efficacy and can be repurposed for additional symptoms, like autoimmune infections, cancer, and viral contaminations [15,22]. Artemisinin-related drugs are also reported to be used for treating antiviral, anticancer, and anti-inflammatory diseases [23].

Artemisinins are considered as prodrugs, due to two possible reasons: firstly, many derivatives are promptly transformed to dihydroartemisinin under in vivo conditions, and secondly, their mechanism of action depends on the stimulation by cleavage of the endoperoxide bridge [21,24]. Prodrugs are usually the precursor compounds that are metabolized in the body and transform into a pharmacologically active drug compound [25]. These prodrugs are used, due to their enhanced pharmacokinetics attributes such as absorption, circulation, breakdown, and defecation [25]. Artemisinin-based drugs are considered liver-activated prodrugs of di-hydro-artemisinin [26,27,28,29]. These prodrugs are reported to be used in the treatment of malaria [15]. Considering all the potential benefits of Artemisinin, in the current review a concise account of the history of the antiviral and antimalarial prodrugs—Artemisinin, from the *Artemisia* sp.—has been discussed, along with its chemical nature, extraction procedure, photochemistry, and clinical uses.

## 2. *Artemisia* sp.: Source of a Powerful “Prodrug”

The term “prodrug” was first used in the year 1951, by Adrien Albert [30]. The prodrug is an inactive compound, and is different from the fully active form of a compound that gets transformed into the active compounds (drugs) by a series of metabolic pathways, by the enzymes like hydrolases or by other chemical actions, or by a combination of the two inside the body of human being [31,32]. These prodrugs are molecules with slight, or without any, pharmacological effect [33]. To exert its full pharmacological functions for targeting various diseases, these prodrugs undergo a series of biochemical alterations [34]. Generally, prodrugs are altered from the parent molecule and selectively show effects in the targeted tissues [32]. Usually, prodrugs are available as bio-precursors or carrier-bound [31,35]. Carrier-bound prodrugs are comprised of a non-toxic carrier that is joined covalently with the active drug [31,36]. The esters, amides, phosphates, carbamates, imine, oximes, carbonates, and N-Mannich, are the carrier-linkers and the chief groups of prodrugs [31,37]. Prodrug approaches are frequently used to overcome the deficits in the molecule’s physicochemical properties, which limits the options of formulation and consequences in undesirable biopharmaceutical performance [33]. Numerous studies have reported that the Artemisinin prodrug is usually found in *Artemisia annua* L. plant; however, there are other plants like *Artemisia absinthium* L., *A. dracunculus* L., *A. pallens* Wall. ex DC., *A. maritima* L., etc., which possess this compound [38]. Usually, Artemisinin, in different concentrations, has been reported to be found in different parts of these plant species. Artemisinin, is a polycyclic sesquiterpene lactone that has an endoperoxide function. The derivatives of artemisinin are comprised of dihydro-artemisinin, artesunate, arteannuin-B, artemether, and artemisone, and are collectively called “artemisinins”. Artemisinin, being a sesquiterpene lactone, is found more in aerial plant parts as compared to the roots [38]. Table 1 summarizes the different plant species that contain the Artemisinin prodrug. These compounds are reported to have numerous activities with substantial pharmacological uses, including the management of rheumatoid arthritis, systemic lupus erythematosus, allergic contact dermatitis, anticancer, and malaria [39,40].

## 3. Chemical Nature, Phytochemistry and Extraction of Artemisinin

**Figure 1:**
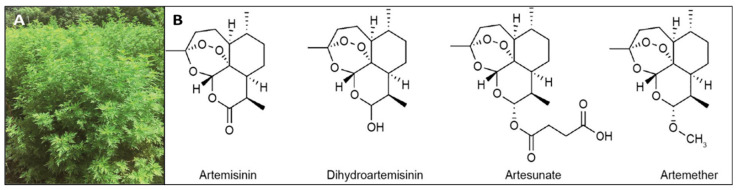
(**A**)—Photo of an *Artemisia* sp. (*Artemisia annua* L.); (**B**) Chemical structure of Artemisinin and its derivatives. Reproduced under a Creative Commons license from Wang et al. (original source Figures 1 and 2) [21].

The unique chemical structure of Artemisinin as a sesquiterpene lactone includes endoperoxide moiety (Figure 1). Artemisinin is less soluble in both water and oil, but is soluble in various aprotic organic solvents [52,53]. Artemisinin can be reduced to dihydroartemisinin using a relatively weak reducing agent such as sodium borohydride in which a hydro reduction reaction transforms the lactone group of artemisinin to a lactol, while conserving the crucial endoperoxide moiety [52,53]. Ether formation and esterification of artemisinin contribute to the stabilization of artemisinin-based drugs [54,55]. Fresh investigations demonstrated that the antimalarial potential of artemisinin-associated compounds is highly superior to that of only Artemisinin [15,56]. The temperature reaches its melting point at around 156–157 °C, by which time also it is highly thermostable [52]. No noticeable decomposition is perceived. When the temperature rises to 190 °C, this molecule starts to break down [52,57]. It is also not stable in the presence of an acid or alkali, which affects the generation of new products [52,58,59]. Numerous compounds have been isolated from the Artemisia sp. recently, and a few of them are presented in Table 2.

**Figure 2:**
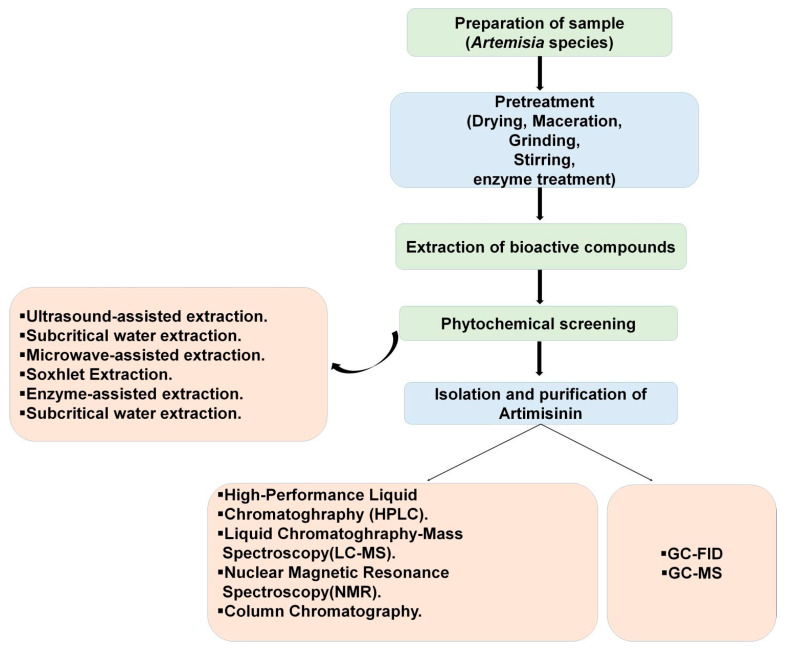
Analysis, extraction procedure, isolation and purification of Artemisinin.

There are various extraction procedures available to extract Artemisinin and its derivatives. Some of the established extraction procedures are discussed below. As discussed by Briars and Paniwnyk [63], about 12.5 g of the dried leaf sample was dissolved in 250 cm^3^ of hexane in a closed flask at different temperatures in a water bath and the samples were analyzed by HPLC at regular time intervals. In the Supercritical CO_2_ extraction procedure, the sample (100 g) was put in the extractor, under 15 bar and 25 °C separation conditions and the extraction of the compound was carried out at 300 bar pressure [64]. Another is the ultrasound-assisted extraction procedure. It can be carried out at different temperatures through an ultrasound bath and for different treatment times. Three solvent/solid ratios like 10, 20, and 30 mL/g can be used with a frequency of 37 kHz and a power of 50 W. Next, the extract needs to be filtered using filter paper and then evaporated [64]. The next procedure is the subcritical water-extraction procedure. In this extraction method, a batch-type high-pressure extractor is used [64]. Further, the Deep Eutectic Solvent Extraction procedure was also reported for the extraction of artemisinin. In this process, the deep eutectic solvents carried out the extraction by the mixing and heating of the sample. [64]. However, there are a few limitations, like the cost of supercritical CO_2_ equipment. Also, there is the possibility of some co-extracted contaminants like pigments [64]. Artemisinin can be extracted by using a microwave-assisted extraction procedure for *A. annua* L. In this extraction process, the period of microwave radiation is 12 min. With the increase in degree of grinding, the extraction rate of artemisinin increases. Among different extraction methods, like supercritical CO_2_ extraction, normal stirring extraction, the Soxhlet method, and the microwave-assisted extraction method for artimisinin extraction, the microwave-assisted extraction method is a better method, since less time is required with this method and the extraction rate is higher than other methods [65]. Figure 2 shows different types of extraction procedures used for the extraction of artimisinin.

### Large-Scale Extraction of Artemisinin

There are a few reports on the large-scale extraction of Artemisinin, some of which are discussed below. Elsohly et al. [66] have described the large-scale extraction of artemisinin, using the leaves of *Artemisia annua*. The author mentioned that, initially, a hexane extract is prepared by taking the ungrounded leaves (for 400 kg) of *A. annua* and mixing them with hexane solvent. Next, the hexane extract was separated with acetonitrile (20%) at a 1:3 ratio, which helped in the quantitative transmission of artemisinin to the aqueous acetonitrile layer. Moreover, the hexane residue was further dissolved in hexane (12 mL/g) and allowed to stand for overnight. It was filtered, the solution was again separated with acetonitrile (20%), and the steps were repeated several times and with different other solvents. Finally, the concentrated form of artemisinin was isolated using the chromatography method on Si gel filtration columns. This method allows the reuse of the recovered solvents and the chromatographic columns [66].

In another study, Zhang et al. [67] have demonstrated the ultrasonic assisted-extraction method for artemisinin. The authors reported that ultrasonic assisted-extraction using monoether-based solvents is an efficient green technique used to separate artemisinin from *Artemisia annua* L. Among the tested monoethers, the performance of propylene glycol methyl ether is best for the extraction of artemisinin. The ideal condition for this extraction is ultrasonic power 180 W, temperature 38 °C, and ultrasonic time 30 min. Consequently, the proposed approach showed higher extraction efficiency and significantly reduced extraction time and temperature, and decreasing energy consumption. In addition, minor toxicity of the propylene glycol methyl ether solvent and reduced vapor pressure made the extraction process safer and more reliable [67].

In 2019, Rodrigues et al. [68] reported on the industrially and economically feasible technique of extracting artemisinin from the leaf powder of *A. annua*. Optimized conditions for temperature, pressure and the various solvents used in the supercritical fluid extraction were evaluated and compared to the conventional extraction procedures. The authors showed that an optimized condition of supercritical fluid extraction at 60 °C and 250 bar, without using any co-solvent, was the most favorable extraction condition. However, for the marketable feasibility of the extraction of arteminisin using supercritical fluid extraction, the use of specifically selected varieties of *A. annua* plants is specified. Recently, Babacan et al. [69] have reported on the extraction of artemisinin from *A. annua* plants using the high-performance liquid chromatography method optimized using response surface methodology. In the extraction design, powdered charcoal and ethanol were chosen as the best extraction adsorbent and solvent, correspondingly. Artemisinin was collected from the solvent in the form of concentrated crystallized artemisinin [69]. According to the authors, the optimized extraction protocol developed by them can be used as a new and green alternative for the industrial-scale or large-scale extraction of artemisinin from A. annua samples [69]. A recent review article has also been published on the large-scale production strategies of artemisinin and its advancements, along with the effect of the environment on the cultivation of the *A. annua* plant [70].

## 4. Artemisinin: Its Antiviral Prospective

For centuries, *Artemisia* sp., (*A. annua* and *A. afra*) have been used in the treatment of several health conditions. Whereas medicinally, artemisinin is the most effective element, *Artemisia* sp. from diverse subgenera with an antiviral effect are widespread, and are the most important antiviral species belonging to the subgenus *Artemisia* [71]. *Artemisia* sp. have been used for centuries to treat various diseases. As per an earlier report, all the phytochemicals including coumarins, flavonoids, phenolic acids, and other terpenes found in this genus are also medicinally effective. The secondary metabolites present in *Artemisia* sp. improve the bioavailability of artemisinin, thereby enhancing the medicinal potential of the compound synergistically [72]. *A. annua* L. has an earlier history in treating fever, which is a common sign of various contagious ailments, as well as viruses [73]. During the 1980s, Chinese scientists highlighted the probable antiviral effect of artemisinin [74]. Artesunate (a semi-synthetic derivative of artemisinin) has been reported to hinder the duplication of human cytomegalovirus and the herpes simplex virus type 1 [75]. *Artemisia* sp., like *A. annua* and *A. afra*, showed positive effects against COVID-19, and SARS-CoV-2 infection and the associated symptoms [76]. The current literature has suggested the efficacy of Artemisia or artemisinin derivatives in combatting SARS-CoV-2 infection, by reducing oxidative stress and inflammation, and in mitigating lung injury [76]. There is a report that artemisinin and its derivatives displayed good bioavailability, as well as good oral absorption. It also confirmed its ethnopharmacological use and probable antiviral action against the COVID-19 virus [77].

The screening of *Artemisia* extracts showed that derivatives like artemisinin, and whole aerial part extracts of Artemisia sp. displayed virucidal effects against various types of viruses, like Corona viruses [78,79]. It is also reported that extract from different species of *Artemisia genus* displayed a strong inhibitory effect by means of controlling inflammation and the immune response [79,80,81]. In *Artemisia* sp., a number of studies have also supported the existence of anticoagulant particles [79,82,83,84]. In numerous research studies into a varied range of DNA and RNA viruses, the broad-spectrum antiviral prospective of artemisinin has been established [40].

In a study by Romero et al. [85], the antiviral property of artemisinin from *A. annua* against a bovine viral diarrhea virus has been reported. The author reported that artemisinin, in combination with ribavirin, decreased the viral-prompted cell death by causing a substantial decrease in the manufacture or release of the bovine viral diarrhea virus by the infected EBTr cells [85]. Further, in a study by Karamoddini et al. [1], the authors have reported the positive antiviral effects of the subset extracts from the aerial parts of *Artemisia* sp., such as *A. annua*, *A. campesteris*, *A. chamaemelifolia*, *A. fragrans*, *A. incana*, *A. persica*, and *A. vulgaris*, against the Herpes Simplex type I virus. Reports say that artemisinins are able to block the tissue fibrosis, and act as anti-inflammatory and antioxidant agents with less toxicity, making them excellent drug aspirants against the Coronavirus infection [2,86]. Some other in vitro studies have reported on the inhibitory effects of artemisinin against the hepatitis B virus [87]. Zhou et al. [88] have studied the efficiency of artemisinin against SARS-CoV-2, and found that the treatment with all studied extracts and compounds inhibited SARSCoV-2 infection of VeroE6 cells, human hepatoma Huh7.5 cells, and human lung cancer A549-hACE2 cells, without any noticeable impact of the cell type on antiviral efficacy [88]. There are reports on the potential effect of artemisinin hindering the capacity of COVID-19, by affecting the ability of the virus to proliferate [89]. Several imperative clinical trials for the cure of the COVID-19 pandemic have been reported worldwide, such as the two artemisinin-related studies recorded in ClinicalTrials.gov.: Artesunate (NCT04387240) and ArtemiC (NCT04382040) [90]. Also, a new patent was published in 2020, appealing for the treatment of COVID-19 infections by using herbal formulations with *A. annua* (CN111150755) [90].

## 5. Artemisinin: Its Antimalarial Prospective

In spite of progress in the medical field, malaria is still considered as one of the universal health issue that causes approximately more than 1 million deaths per year, worldwide [91], and more than 1 billion people live in malaria-affected areas [92]. Recently, in 2022, globally, malaria cases accounted for about 249 million, which is well above the projected number of cases before the COVID-19 pandemic, and an upsurge of 5 million cases over the previous year [93]. In 2022, an estimate of around 1 million deaths has been reported globally, due to malaria, with a mortality rate of 14.3 deaths/100,000 population to be the estimated risk [93]. Previous reports have claimed that the use of artemisinin in the treatment of malaria dates back to 1972, when a Chinese scientist identified artemisinin as the active compound of *A. annua* while researching a new antimalarial drug [94]. In 2006, the WHO endorsed the implementation of ABCT as a first-line of action for the management of *Plasmodium falciparum* malaria [95,96], and this treatment procedure has also been used for malaria treatment [40,97]. In addition, since then, different semi-synthetic derivatives of artemisinin, like arteether, artesunate, artemether and dihydroartemisinin, have been utilized in the treatment of malarial infection [95,96,98]. Since then, artemisinin-based combination-therapy treatment has been considered as an effective way to treat and reduce the transmission rate of malaria [22,96]. Moreover, their high safety and acceptability adds to their desirability as antimalarial drugs, worldwide [96,99]. Artemisia herbs are comprised of artemisinin and its derivatives, including many bioactive molecules that were efficiently used to treat malaria [79,100,101]. Reports have claimed that clinical trials of artesunate alone used dosages of 1–8 mg/kg intravenously or 0.6–1.2 g/day orally for five days of treatment, whereas, in combination, 4–25 mg/kg intravenously or 0.2–0.8 g/day orally for three days, have been used [96,102]. As per a review article on the safety of artemisinin, it is reported that the use of artemisinin-based treatment in malaria is considered as safe and without any side effects and toxicity effects [103]. In a recent study, Tripathi et al. [104] have studied the transcriptome, drug-sensitivity profile, and cellular ultrastructure of *P. falciparum* parasites treated with artemisinin combination treatments. Their results suggested that persistent forms of *P. falciparum* parasites might deliver a reservoir for recrudescent infection after artemisinin monotherapy and, thus, play a significant part in the failure of artemisinin combination treatments [104]. In one study, Liu et al. [105] have demonstrated the therapeutic potential of artemisinin in rodent Polycystic ovarian syndrome -like models and human patients with Polycystic ovarian syndrome by assessing the artemisinin-derivative effect on testosterone level, estrous cycle, and polycystic ovarian morphology.

## 6. The Rise of Specific Artemisinin Resistance in Malaria, Particularly in Southeast Asia

In the control of malaria worldwide, partial resistance to artemisinin in *Plasmodium falciparum* is one of the most persistent horrors. Across Southeast Asia, partial resistance to artemisinin has spread widely over the last two decades, affecting public health schemes and delaying eradication efforts. Some previously published articles have reported on the critical role of genomic surveillance, and how it will help in combating the spread of partial resistance to artemisinin [106]. Further, it will be critical to constantly develop and secure the use of genomic investigation as a vital part of the observation toolkit for artemisinin partial resistance to artemisinin, which comprises approaches like treatment-effectiveness education and in vitro phenotypic educations. An upsurge in partial resistance to artemisinin may be identified without coordinating these surveillance tools, and only when it has already spread widely, adversely affecting patient outcomes and compromising our capability to implement mitigation approaches [106,107,108]. Hence, enduring investigation is vital, not only for compressing the spread of partial resistance to artemisinin, but also for reducing the human cost it will generate [106].

The current occurrence of artemisinin resistance in regions like in Southeast Asia presents a serious contest in the fight against malaria. To fight against this emerging challenge, a complete understanding of the pathways involved in mediating resistance is essential. In artemisinin resistance, mutation in the *PfKelch13* gene has been confirmed as a marker. In conferring resistance, it is not astonishing to find the involvement of numerous pathways. The functional role of the *PfKelch13* gene has been revealed in earlier studies: it possess two distinct roles, i.e., one is that of regulating host hemoglobin endocytosis, and the other is as an ubiquitin ligase regulator. Its detailed role and degree of its multifunctionality are still unidentified. Future investigation needs to investigate the complicated crosstalk which exists between metabolism and gene expression by epigenetic mechanisms. As artemisinin resistance is linked with metabolic renewing, the gene-expression alterations might occur by the action of metabolites and epigenetic regulators. Thus, sustained investigation into understanding the molecular and metabolic pathways of resistance holds potential for the improvement of advanced interventions for deactivating artemisinin resistance and adds to the constant efforts in the control and elimination of malaria [109].

## 7. Mode of Action of Artemisinin

A number of mechanisms of action of artemisinin as an antiviral and antimalarial prodrug have been explained recently [21]. During the 1980s, the Chinese scientists were the first to provide information on the antiviral potential of artemisinin and its possible mode of action [74]. As suggested, artesunate repressed the central regulatory processes of human cytomegalovirus-infected cells, thus interfering with the critical host-cell type and metabolism requirements for the replication of human cytomegalovirus [96]. Further, it was reported that the anticytomegaloviral activity of artesunate was not confined to human cytomegalovirus only, but also included the animal cytomegalovirus, such as the rat cytomegalovirus [110]. In a study by Fillebeen et al. [111], hemin, an iron donor, prevents the replication of the Hepatitis C virus replicon by hindering the viral polymerase, and the combination of artemisinin and hemin treatment could have a noticeable synergistic antiviral effect without disturbing host cells.

Just recently, the world has overcome a severe encounter with the COVID-19 pandemic (a viral infection), which has affected global health, causing maximum distress. Earlier, several in vitro studies have specified that artemisinin or its derivatives could have potential antiviral effects against a number of virus, including the Herpesviridae family [10,96,112].

**Figure 3:**
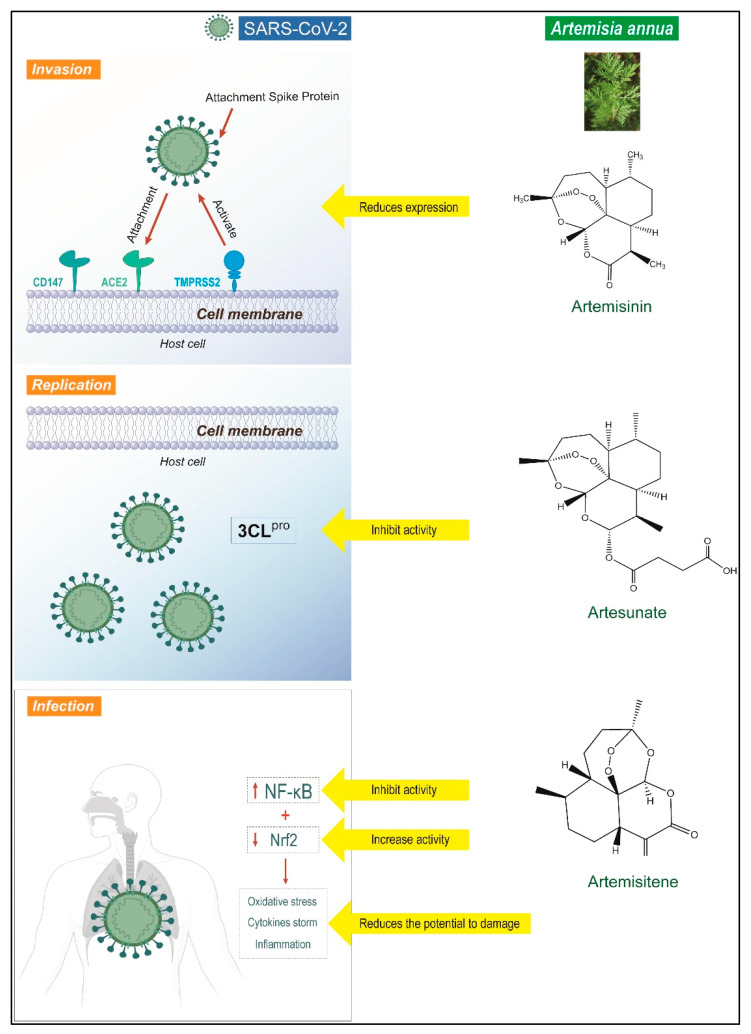
A possible mode of action of *A. annua* L. and its derivative compounds against the corona virus infection (SARS-CoV-2). Reproduced under the terms of Creative Commons Attribution-Noncommercial 4.0 License (CC BY-NC 4.0), permitting all non-commercial use, distribution, and reproduction in any medium, from Ahmad et al. [114].

A study by Zhou et al. [88] has reported the effect of ABCT in fighting the SARS-CoV-2 virus infection, and has stated that the possible mode of action of artesunate could be by attacking the virus at its post-entry level. In another study, the author stated that the dried-leaf hot-water extracts of *A. annua* cultivars displayed in vitro anti-SARS-CoV-2 activity against all variants of the virus, including the original wild type [113]. Similarly, in a review article by Ahmad et al. [114], the authors have discussed the beneficial properties and probable antiviral mode of actions of *A. annua* L. and its associated compounds against SARS coronavirus contamination. The author stated that *A. annua* might act against the SARS-CoV-2 infection by a number of possible mechanisms, such as through the angiotensin-converting enzyme, virus replication, hindering its invasion, cluster differentiation, and transmembrane protease serine 2 expression, decreasing oxidative stress and inflammation by weakening the Nrf2 and NF-kB signaling, and extenuating lung damage in patients infected with the COVID-19 virus (Figure 3) [114].

**Figure 4:**
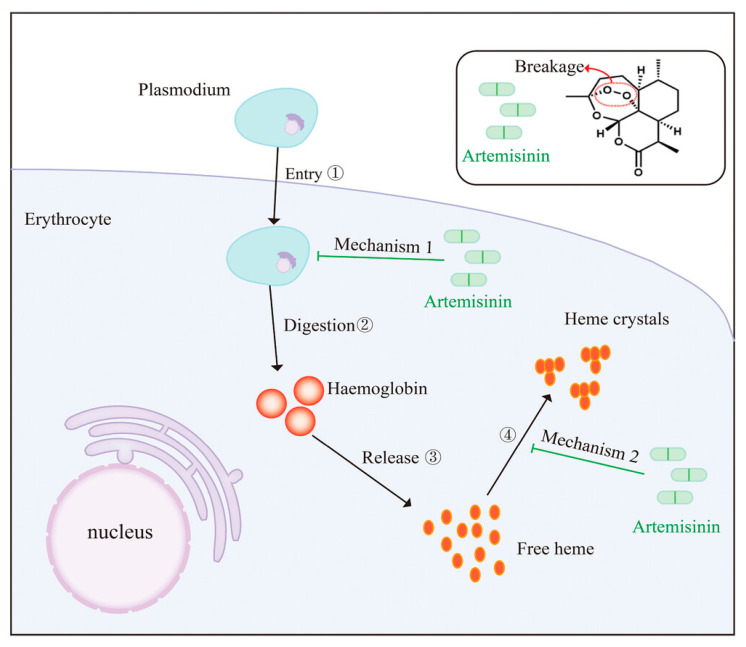
A possible mode of antimalarial action of artemisinin-based drugs. Reproduced with permission from Guan et al. (original source Figure 2) [15].

More than ten years have passed since the use of artemisinin-based malarial treatment, and since then a number of clinical and pharmacological features of artemisinin therapy have been widely studied and reported [115,116,117]. Though the nature of different derivatives can change, artemisinin-based drugs are categorized by their rapid action and effectiveness, less toxicity, and a short half-life, which makes artemisinin-based combination treatment favorable for antimalarial drugs [117]. Generally, the mechanism of action of artemisinin can be thought of as a result of its exclusive mode of activation, and its drug targeting [21]. The antimalarial activity of Artemisinin has been reported to be due to its endoperoxide bridge, which, upon contact with the abridged heme or ferrous iron in the parasite, forms free radicals that alkylate the vital plasmodial biomolecules, resulting in the death of the malarial parasite [95,118,119]. Several studies have shown that artemisinin and its derivatives could have acted by inducing the Plasmodium’s mitochondrial and plasma membrane depolarization, which is linked to the reactive oxygen species [120,121]. In the year 2023, another interesting review article discussed the mode of antimalarial action of artemisinin-based combination drugs [15]. The author stated that the pathogen *Plasmodium* digests the hemoglobin from the human blood in the erythrocytic phase of its life cycle, and isolates the resultant toxic free heme as nontoxic heme crystals; further, these freshly released free hemes stimulate the carbon-centered radical of artemisinin and then alkylates the proximal protein, and then kills it [15]. Another mode of action is for the stimulated artemisinin to alkylate the heme, making the heme drug adducts that prevent the creation of heme crystals, which leads to the killing of the pathogen (Figure 4) [15].

## 8. Novel Perspectives of Artemisinin Hybrid Compounds and Their Biological Activity

Artemisinin has some deficiencies, which include a short half-life, poor solubility and restricted bioavailability, and, in order to overcome these deficiencies, the hybridization of this compound with other compounds is very helpful [122]. The hybridization technique can not only prolong the half-life or increase the solubility and bioavailability, but can also diminish the side effects, boost the activity, and counter the drug-resistance [122,123,124]. Hybrid molecules, in which the two compounds are connected, have evolved as an advanced approach within the medicinal chemistry and drug discovery sector, which delivers agents more effectively and accurately [125]. Molecular hybridization is an up-to-date approach used in the discovery of modern drugs with enhanced potential [123]. On the basis of the covalent combination of pharmacophoric moieties of various bioactive molecules, the molecular hybridization process yields new types of hybrid composites which are more effective than the parent compounds [123,126]. Nowadays, the synthesis of hybrid and dimer derivatives of different bioactive compounds has turned out to be a useful approach to increase both the biological activity and pharmacokinetic profiles of various compounds, evading their drug-resistance effects, low toxic side effects, and improving the safety profile [127,128,129]. Some of the hybrid compounds demonstrated noteworthy antimalarial and anticancer properties under both in vitro and in vivo conditions [130].

**Figure 5:**
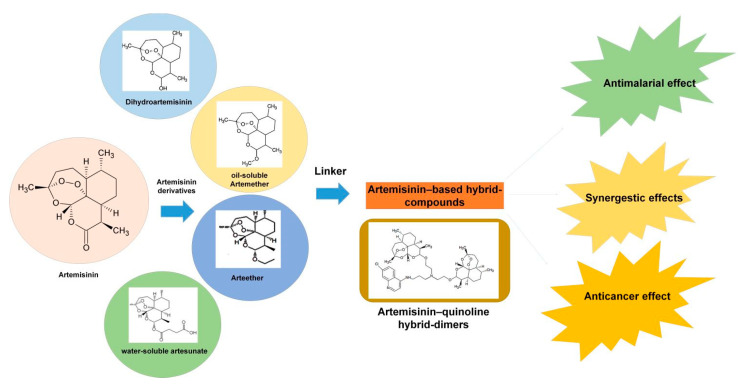
Structure of artemisinin and its derivatives forming artemisinin-based hybrid dimers for various biological activities.

The artemisinin group of compounds comprises artemisinin from *A. annua*, and its semi-synthetic derivatives (reduced lactol, dihydroartemisinin, the oil-soluble artemether, arteether and the water-soluble derivative artesunate) [131]. With regard to artemisinin, alteration to the lactone functionality is well endured with derivatives such as dihydroartemisinin, oil-soluble artemether, arteether and water-soluble derivative artesunate, all possessing potent antimalarial activity (Figure 5) [132]. As dihydroartemisinin, encompassing the simply esterifiable hemiacetal functionality, it is one of the major artemisinin metabolites formed under in vivo conditions. It was designated as the utmost suitable artemisinin derivative to make the artemisinin–quinine hybrid [133]. Dimers and hybrids of artemisinin and artemisinin derivatives, in combination with a number of bioactive compounds like thymoquinone, have been reported to exhibit augmented antileukemia and antimalarial activity [134]. Further, several dimers and hybrids of artemisinin derivatives comprising natural phenol and catechol residues, like 2-(3-hydroxyphenyl)ethanol and 3-hydroxytyrosol, have been patented [135].

The synthesis of hybrid artemisinin–quinine derivatives has been proposed by Walsh, et al. [133]. In the first step, the transformation of the alkene functionality of quinine into its carboxylic acid derivative is involved [136]. Briefly, the hydroxy functionality of quinine was secured as a tert-butyldimethylsilyl ether, consuming Triethylamine, 4-Dimethylaminopyridine and tert-Butyldimethylsilyl chloride. Alteration of the vinyl functionality of tert-butyldimethylsilyl quinine to the carboxylic acid derivative required a hydroboration step with borane/tetrahydrofuran in diglyme and cleavage of the borane complex with Trimethylamine N-oxide dehydrate to produce the primary alcohol which, after oxidation with Jones reagent, afforded the acid. Dihydro-artemisinin was found by the reduction of artemisinin with Sodium borohydride in methanol, and was attached to quinine in its carboxylic acid derivative, using 2,6-dichlorobenzoyl chloride as the coupling reagent, Triethylamine as the base, and 4-Dimethylaminopyridine as the acylation catalyst [133]. The most important hybrid isomer was purified by thin-layer chromatography [133].

In another study, Lombard et al. [137] have described the synthesis of novel artemisinin–quinoline hybrid dimers from dihydro-artemisinin and different aminoquinolines, at an higher temperature of 90–110 °C. All compounds were found as β-isomers, and were tested for both chloroquine-sensitive and -resistant strains of *P. falciparum*. The results obtained showed that the hybrid dimer 8 has the uppermost antiplasmodial activity [137].

Lombard et al. [138] described the promising antimalarial activity of the two artemisinin–quinoline hybrid dimers in a rodent model, and concluded that the hybrid dimers exhibited significant effect against in vitro proliferation of the tumor cell lines.

Joubert et al. [139] have synthesized an artemisinin–acridine hybrid (2-Bromo-(10β-dihydroartemisinoxy)ethane) and studied its antiplasmodial activity. It was reported to be sevenfold more active than chloroquine against the *P. falciparum* NF54 strain.

Tien et al. [140] have reported on the synthesis of fourteen new hybrids of propargyl-substituted derivatives and artemisinin containing a 2-hydroxypropane unit, by the click chemistry process. The authors have evaluated the anticancer effects of these compounds and have found that four triazole–artemisinin derivative hybrids are highly effective against two human cancer cell lines, the KB and HepG2 cell lines [140].

Similarly, in another study, the artemisinin–estrogen hybrid displayed better antiplasmodial activity than standard the drugs, against the 3D7 strain [141].

In another study, Frohlich et al. [142] have prepared five artemisinin–quinazoline hybrids and evaluated the biological potential against the malarial parasites (*P. falciparum* 3D7), leukemia cells (CCRF-CEM and CEM/ADR5000), and the human cytomegalovirus. All the hybrid compounds displayed promising activities [142].

Hu et al. [143] have reported the synthesis of a series of artemisinin derivatives with multidrug-resistance reversal activity and anticancer properties. The detailed results showed that most of the new compounds displayed greater antiproliferative activities against the human cancer cell lines (MCF-7, A549 and HepG-2 cells) than the parent artemisinin compound and lower cytotoxicity effect on the normal human hepatic cell cells than the positive control (adriamycin) [143].

Botta et al. [127] have studied the synthesis of a library of hybrids and dimers based on artemisinin. The synthesis of these compounds was carried out by the process of the coupling of artemisinin derivatives, artesunate, and dihydroartemisinin with a series of phytochemical compounds, and studied their anticancer properties against the cancer cell lines [127]. Out of the synthesized compounds, two hybrid compounds formed by the coupling reaction between artesunate with eugenol and tyrosol, and one of the dimer compounds comprising curcumin, displayed the most effective results against the cancer cell lines.

Recently, Vamvoukaki et al. [144] have studied the synthesis and antiplasmodial assessment of hybrids combining the pharmacophore structures of artemisinin, ciprofloxacin, and 7-chloroquinoline. The authors concluded that the combination of artesunate with either ciprofloxacin or norfloxacin moieties in a single molecular entity has been demonstrated to significantly improve the action and selectivity when compared to its unnconjugated counterparts. Taken together, it is found that the artemisinin-based hybrid compounds are proven to be highly effective, with promising antimalarial and other bioactive properties and with fewer side effects, as compared to their parent compounds.

## 9. Clinical Trials and Patents

In clinical trials, Artemisinin and its derivatives have revealed broad-spectrum antitumor effects *in both* in vivo *and* in vitro. From a few number of clinical trials, it is evident that Artemisinin has great antitumor effects. But in the clinic, a few complications, like toxicity, poor solubility, and controversial mechanism of action, hinder its usage as an active antitumor agent [145]. In China, since 1979, artemisinin and numerous derivatives of it have been produced, and research has been started into it. Intramuscular artemether and dihydro-artemisinin, artemisinin suppositories, and artesunate (oral or parenteral) drugs (tablets) have been widely shown to be quickly effective. According to Li et al., in China, these drugs have replaced quinine and chloroquine in the treatment of malaria [146].

The first effective clinical trial of artesunate was carried out for treating human cytomegalovirus in a patient who showed drug-resistant contagion during preventive antiviral therapy after stem-cell transplantation [147]. The triple ABCT, which associates artemisinin and its related compounds with two companion drugs, is efficient and well tolerated for multidrug-resistant malaria. Using *P. falciparum* epidemiology and evolution, it was appraised whether the introduction of each artemether–lumefantrine–amodiaquine or artesunate–mefloquine–piperaquine caused minor long-term artemisinin-resistance levels [108]. There are studies on the treatment of artemisinin and its derivatives in three thousand clinical malarial patients, against drug-resistant *P. falciparum* [148,149]. There are also some comparative studies on the whole plant and the extracts of *A. annua*, along with chloroquine, in treating malaria [150,151]. Moreover, a few human trials have also shown the potential effect of artemisinin–piperaquine against the COVID-19 virus [152,153].

Advantageous artemisinin-based drug combinations and artemisinin derivatives have been established for use in numerous healing purposes. According to experts, due to inherent synthetic challenges, the diversity of semi-synthetic artemisinin and its associate compounds is limited to the same design scheme of changing the fragment of artemisinin in the same state [95]. Using the professional patent database, essential patent bibliographic data about the subjects, including artemisinic acid, arteannuin B, and artemisinin, were investigated and documented. The patent technology life cycle of artemisinin is in a mature stage, and, it has a comparatively great scope for progress.

There has been a constant rise in the number of artemisinin patents during the last 30 years since 1986, when the first patent was published [90]. Countries like China and the United States have received a maximum number of patents for this compound, and are also considered as important markets for artemisinin [90]. As per the earlier research, until 2019, a total of 4594 documents of 1450 patent and simple-patent families (from 1986 to 2019) were studied. A broad patent scenario of artemisinin was observed from the phases of patent ownership, co-patents, technological classes, healing areas, time trends, filing countries, pathways, and citation networks [90]. Associated artemisinin compounds are in progress to enhance the efficacy of ABCT, and to lessen its vulnerability to drug resistance [154]. Numerous patents have been granted on Artemisinin research by a number of agencies like companies and university research institutes, and Table 3 summarizes the top 15 agencies who have applied for artemisinin research [90].

## 10. Conclusions and Future Prospectives

Artemisinin is undoubtedly one of the most capable natural compounds that has been studied during the last two decades. Since its discovery, an intensive determination by the worldwide community has accumulated a picture of a drug with exclusive potential, which makes it almost the perfect antimalarial drug. Earlier pharmacokinetic investigations suggested that artemisinins are mostly processed by liver microsomes. In connection with malaria, artemisinin has the prospective to significantly add to altering the desperate condition that the world is facing. The plant species Artemisia is gaining high acceptance for treating or preventing malaria. There is a necessity to address the controversial query of the well-being of the community by providing strong scientific evidence in relation to the uses of the Artemisia species. Fortuitously, the worth of this compound is not restricted to malaria treatment only; it has been tested against a number of viral infections, including the most recent COVID-19 infection. Paradoxically, in an age where scientists are searching for bioactive compounds with enhanced molecular and cellular target specificity, consciousness of the artemisinin compound is growing. This class of compounds appears to have numerous objectives for curing many diseases. The suppression of diseases like viral diseases and malaria will ultimately require a cohesive approach that comprises the amalgamation of new and old drug treatments, vaccines etc., which could be beneficial for human health.

## Figures and Tables

**Table 1:** Artemisinin distribution in different *Artemisia* sp.

Plant Species	Plant Part Involved	Content	Solvent Used	Technique Used	References
*A. annua*	Trichomes of leaves, stem, inflorescence.	0.01–1.4%	Toluene	GC/MS	[41]
*A. pallens*	Leaves and flowers	0.1031%	Ethanol	LC/MS	[42]
*A. cina*	Shoots	0.0006%	n-hexane	HPLC	[43]
*A. sieberi*	Aerial part	0.2–0.14%	Ethanol		[44]
*A. absinthium*	Whole plant	0.02–0.35%	Petroleum ether		[45]
*A. dubia*	Roots	0.01–0.07%	Toluene		[22]
*A. indica*	Roots	0.01–0.10%	Toluene		
*A. dracunculus* var. *dracunculus* L.	Stem	0.12 ± 0.01%	Toluene		
*A. roxburghiana*	Flower	0.23 ± 0.01%	Toluene		
*A. bushriences*	Flower	0.34 ± 0.02%	Toluene		
*A. moorcroftiana*	Stem	0.8 ± 0.01%	Toluene		[22,46]
*A. vestita*	Roots	(0.04 ± 0.02%	Toluene		[22]
*A. sieversiana*	Stem	0.8 ± 0.03%	Toluene		[46]
*A. campestris* subsp. *glutinosa*	Aerial parts	0.64%	n-hexane		[47]
*A. herba-alba*		0.34%	n-hexane		
*A. vachanica*		0.34%	n-hexane		[48]
*A. makrocephala*		0.20 ± 0.01%	Hexane		
*A. parviflora*	Leaves	0.87 ± 00.2%	Toluene	RP-HPLC RP-HPLC	[49]
*A. myriantha*	Leaves	0.039%	Toluene		
*A. japonica*	Leaves and florets	0.4–1.3 mg^−1^ dry wt.	Ethyl acetate and methanol	HPTLC	[50]
*A. absinthium*	Whole plant	2.32 ± 0.02%	Ethanol	LCMS	[51]
*A. maritima*		1.26 ± 0.03%			
*A. dracunculus*		0.78 ± 0.02%			
*A. verlotiorum*		1.11 ± 0.03%			
*A. vestita*		1.06 ± 0.03%			
*A. vulgaris*		2.18 ± 0.03%			

Source: Reproduced with permission from Nabi et al. (original source Table 2) [38].

**Table 2:** Secondary metabolites isolated from *Artemisia* species in the previous decade.

Plant Name	Plant Part	Bioactive Compound	References
*Artemisia annua*	-	Artemanin A	[60]
	-	Artemanin B	
	-	Eriodictyol-7-O-hexoside	
	-	Caffeoylcoumaroyltartaric acid	
	-	Artemin	
	Flowers, Leaves, stems	Artemisinin	[61,62]
*A. dracunculus* var. *dracunculus* L.	Leaves, Stems		[9,22]
*A. moorcroftiana*	Stems		
*A. parviflora*	Stems		
*A. sieversiana*	Stems		

**Table 3:** Selected number of assignees for obtaining patents on artemisinin.

Rank	No. Patent Documents	Patent Families	Average No. of Patents per Family	Assignees	Assignee Type
1	56	55	1.02	Yuzhou City Tianyuan Biological Technology (China)	company
2	78	49	1.59	Shanghai Jiao Tong University (China)	university and research institute
3	164	42	3.9	Council of Scientific and Industrial Research (India)	university and research institute
4	47	17	2.76	Kunming pharmaceutical corporation (China)	company
5	81	16	5.06	Johns Hopkins University (U.S.)	university and research institute
6	81	15	5.4	University of Washington (U.S.)	university and research institute
7	14	11	1.27	Institute of Chinese Materia Medica, China Academy of Chinese Medical Sciences (China)	university and research institute
8	14	11	1.27	Guilin Nanyao Pharmaceutical (China)	company
9	140	10	14	Centre national de la recherche scientifique (France)	university and research institute
10	48	10	4.8	Dafra Pharma International (Belgium)	company
11	228	9	25.33	Sanofi S.A. (France)	company
12	92	9	10.22	Medicines for Malaria Venture (Switzerland)	university and research institute
13	63	9	7	University of California (U.S.)	university and research institute
14	19	9	2.11	Shenyang Pharmaceutical University (China)	university and research institute
15	8	8	1	Ocean University of China (China)	university and research institute

Source: Reproduced with permission from Liu et al. (original source Table 1) [90].
